# Romberg extrapolation for Euler summation-based cubature on regular regions

**DOI:** 10.1007/s13137-017-0097-4

**Published:** 2017-09-11

**Authors:** W. Freeden, C. Gerhards

**Affiliations:** 1 Geomathematics Group, University of Kaiserslautern, 67653 Kaiserslautern, Germany; 2 Computational Science Center, University of Vienna, Oskar-Morgenstern-Platz 1, 1090 Vienna, Austria

**Keywords:** Cubature, Romberg extrapolation, Euler summation, 65D30, 65B99

## Abstract

Romberg extrapolation is a long-known method to improve the convergence rate of the trapezoidal rule on intervals. For simple regions such as the cube $[0,1]^q$ it is directly transferable to cubature in *q* dimensions. In this paper, we formulate Romberg extrapolation for Euler summation-based cubature on arbitrary *q*-dimensional regular regions $\mathcal {G}\subset \mathbb {R}^q$ and derive an explicit representation for the remainder term.

## Introduction

Numerical integration methods are of crucial importance in various applications. Among the first methods were Newton-Cotes formulas on intervals, with the trapezoidal rule being a specific example. In Romberg’s paper Romberg (1955), an iterative superposition of the trapezoidal rule for different grid sizes $\tau$, $\frac{1}{2}\tau$, $\frac{1}{4}\tau , \ldots , \frac{1}{2^\ell }\tau$, was introduced to improve the convergence rate for the approximation of the one-dimensional integral $\int _0^1 F(y)dy$ from originally $\mathcal {O}(\tau ^2)$ to $\mathcal {O}(\tau ^{2\ell })$ (depending on the number $\ell$ of iterations and the smoothness of the integrand *F*). This method can be directly transferred from the interval [0, 1] to the *q*-dimensional unit cube $[0,1]^q$ (see, e.g., Antes 1972; Schönhage 1970). More advanced methods came into play with a thorough study of lattice rules (see, e.g., Freeden 2011; Lyness 1989; Sloan and Joe 1994 for overviews). Also quasi-Monte Carlo became popular, which leaves the path of fixed lattices but chooses cubature nodes in such a way that they yield ’good’ convergence of an equal-weight cubature rule (see, e.g., Caflisch 1998; Dick et al. 2013 for overviews). An exhaustive overview on various construction principles for cubature rules is presented in Cools (1997). However, those studies are mostly restricted to standard regions like the unit cube.

In many applications integrals have to be computed over more complicated regions $\mathcal {G}\subset \mathbb {R}^q$. In this paper, we focus on Euler summation-based cubature rules on such regions $\mathcal {G}$, as deduced, e.g, in Freeden (1982), Freeden and Fleck (1987). Investigations that are restricted to $[0,1]^q$ often camouflage the problems associated with general integration regions $\mathcal {G}$. Let us take, e.g., a lattice rule

1.1 $$\begin{aligned} Q_\tau [F]={\sum _{g\in \tau \mathbb {Z}^q\cap \overline{\mathcal {G}}}}w_g F(g), \end{aligned}$$

where $\mathbb {Z}^q$ denotes the underlying lattice and $w_g$ the cubature weights, as an approximation for

1.2 $$\begin{aligned} \int _{\mathcal {G}}F(y)dy. \end{aligned}$$

If $\mathcal {G}=[0,1]^q$, the trapezoidal rule is of the form (1.1) and yields $|Q_\tau [F]-\int _{\mathcal {G}}F(y)dy|=\mathcal {O}(\tau ^2)$, and Romberg extrapolation maintains the form (1.1) and improves the convergence to the previously mentioned rate $\mathcal {O}(\tau ^{2\ell })$. But for general regions $\mathcal {G}$ this is not the case. Boundary integrals over $\partial \mathcal {G}$ have to be included in the cubature rule $Q_\tau$ in order to obtain the desired convergence rate. However, these boundary integrals are no peculiarity suddenly arising in higher dimensions or for general regions $\mathcal {G}$. They are rather an ingredient to any cubature rule, only that in simple cases like intervals and cuboids the boundary integrals are reduced to point evaluations, so that the cubature rule takes the form (1.1). The goal of this short paper is to provide an explicit formula for the structure of these boundary integrals for general $\mathcal {G}\subset \mathbb {R}^q$ and to formulate a corresponding Romberg extrapolation method.

In Sect. 1.1, we illustrate the one-dimensional case while in Sect. 2, we recall some foundations of Euler summation on regular regions and their connection to numerical integration. In Sect. 3, we then apply Romberg extrapolation to these representations and derive an explicit representation of the remainder term.

### The 1-D case

We start by defining the auxiliary functions

1.3 $$\begin{aligned} G(\Delta ;x)&=-\frac{(x-\lfloor x\rfloor )^2}{2}+\frac{x-\lfloor x\rfloor }{2}-\frac{1}{12},\quad x\in \mathbb {R}{\setminus }\mathbb {Z}, \end{aligned}$$

1.4 $$\begin{aligned} G(\Delta ^2;x)&=-\frac{(x-\lfloor x\rfloor )^4}{24}+\frac{(x-\lfloor x\rfloor )^3}{12}-\frac{(x-\lfloor x\rfloor )^2}{24}+\frac{1}{720},\quad x\in \mathbb {R}{\setminus }\mathbb {Z}, \end{aligned}$$

which satisfy $\Delta G(\Delta ;x)=\Delta ^2G(\Delta ^2;x)=-1$, $x\in \mathbb {R}{\setminus }\mathbb {Z}$ and are arbitrarily often piecewise continuously differentiable. In the one-dimensional setting, $\Delta$ and $\Delta ^2$ simply denote the second- and fourth-order derivatives $\frac{\partial ^2}{\partial x^2}$ and $\frac{\partial ^4}{\partial x^4}$, respectively, whereas $\nabla$ and $\nabla ^3$ denote first- and third-order derivatives $\frac{\partial }{\partial x}$ and $\frac{\partial ^3}{\partial x^3}$, respectively. Using integration by parts over the interval [0, *b*] and choosing *n* such that $\tau =\frac{b}{n}$, for some $\tau >0$, we obtain for a function $F\in C^{(4)}([0,b])$,

1.5 $$\begin{aligned}&\tau ^4\int _{[0,b]}G(\Delta ^2;\frac{y}{\tau })\Delta ^2F(y)dy\nonumber \\&= Q_\tau [F]-\int _{[0,b]}F(y)dy+\tau ^2\left[ \left( \Delta G(\Delta ^2;\frac{y}{\tau })\right) \nabla F(y)\right] _{y=0}^{y=b}\nonumber \\&\quad -\tau ^3\left[ \left( \nabla G(\Delta ^2;\frac{y}{\tau })\right) \Delta F(y)\right] _{y=0}^{y=b}+\tau ^4\left[ G(\Delta ^2;\frac{y}{\tau })\nabla ^3F(y)\right] _{y=0}^{y=b}\nonumber \\&= Q_\tau [F]-\int _{[0,b]}F(y)dy\nonumber \\&\quad -\frac{\tau ^2}{12}(\nabla F(b)-\nabla F(0))+\frac{\tau ^4}{720}(\nabla ^3 F(b)-\nabla ^3 F(0)), \end{aligned}$$

where

1.6 $$\begin{aligned} Q_\tau [F]=\tau {\sum _{k=0,\ldots ,n}}'F(k\tau )=\tau {\sum _{k=1,\ldots ,n-1}}F(k\tau )+\frac{\tau }{2}F(0)+\frac{\tau }{2}F(b). \end{aligned}$$

Latter is the classical one-dimensional trapezoidal rule and (1.5) yields the corresponding error estimate $\big |Q_\tau [F]-\int _{[0,b]}F(y)dy\big |=\mathcal {O}(\tau ^2)$. For the first Romberg step ${Q}_{c,\tau }=\frac{Q_{c\tau }-c^2Q_\tau }{1-c^2}$, for some $c\in (0,1)$, it can be easily checked that the convergence rate of the cubature rule improves to $\big |Q_{c,\tau }[F]-\int _{[0,b]}F(y)dy\big |=\mathcal {O}(\tau ^4)$. This is what we want to generalize to higher dimensions and regular regions $\mathcal {G}\subset \mathbb {R}^q$.

## Euler summation-based cubature

We briefly recapitulate results on Euler summation and numerical integration as can be found, e.g., in Freeden (1982, 2011), Freeden and Fleck (1987), Freeden and Ostermann (2013). First, some basic definitions are required. If not mentioned otherwise, the dimension *q* is always assumed to be $q\ge 2$.

### Definition 2.1

For *q* linearly independent vectors $v^{(1)},\ldots ,v^{(q)}\in \mathbb {R}^q$ the set

$$\begin{aligned} \Lambda =\left\{ g\in \mathbb {R}^q:g=\sum _{i=1}^qa_iv^{(i)}, a_i\in \mathbb {Z},i=1,\ldots ,q\right\} \end{aligned}$$

is called *lattice* with basis $v^{(1)},\ldots ,v^{(q)}\in \mathbb {R}^q$. The *fundamental cell* corresponding to $\Lambda$ is given by

$$\begin{aligned} \mathcal {F}_{\Lambda }=\left\{ g\in \mathbb {R}^q:g=\sum _{i=1}^qa_iv^{(i)}, a_i\in \left[ -\frac{1}{2},\frac{1}{2}\right) ,i=1,\ldots ,q\right\} . \end{aligned}$$

The *inverse lattice* $\Lambda ^{-1}$ denotes the set

$$\begin{aligned} \Lambda ^{-1}=\left\{ h\in \mathbb {R}^q:h\cdot g\in \mathbb {Z}\text { for all }g\in \Lambda \right\} . \end{aligned}$$

### Definition 2.2

Let $\Lambda$ be a lattice in $\mathbb {R}^q$. A function $G(\Delta ;\Lambda ;\cdot ):\mathbb {R}^q{\setminus }\Lambda \rightarrow \mathbb {R}$ is called $\Lambda$-*Green function* with respect to the Laplace operator $\Delta$ if it satisfies the following conditions:

(i) For all $x\in \mathbb {R}^q{\setminus } \Lambda$ and $g\in \Lambda$, it holds that $G(\Delta ;\Lambda ;x)=G(\Delta ;\Lambda ;x+g)$.
(ii) $G(\Delta ;\Lambda ;\cdot )$ is twice continuously differentiable in $\mathbb {R}^q{\setminus } \Lambda$ and
  $$\begin{aligned} \Delta G(\Delta ;\Lambda ;x)=-\frac{1}{\Vert \mathcal {F}_\Lambda \Vert }, \quad x\in \mathbb {R}^q{\setminus }\Lambda . \end{aligned}$$
(iii) For $|x|\rightarrow 0$, it holds that
  $$\begin{aligned} G(\Delta ;\Lambda ;x)=\left\{ \begin{array}{ll} -\frac{1}{2\pi }\ln (|x|)+\mathcal {O}(1),&{}q=2,\\ \frac{1}{2-q}\Vert \mathbb {S}^{(q-1)}\Vert ^{-1}|x|^{2-q}+\mathcal {O}(|x|^{3-q}\ln (|x|)),&{}q=4,\\ \frac{1}{2-q}\Vert \mathbb {S}^{(q-1)}\Vert ^{-1}|x|^{2-q}+\mathcal {O}(|x|^{3-q}),&{}q\in \mathbb {N}{\setminus }\{1,2,4\},\end{array}\right. \end{aligned}$$
  and that
  $$\begin{aligned} \nabla G(\Delta ;\Lambda ;x)\!=\!\left\{ \begin{array}{ll} -\frac{1}{2\pi }\nabla \ln (|x|)+\mathcal {O}(1),&{}q=2,\\ \frac{1}{2-q}\Vert \mathbb {S}^{(q-1)}\Vert ^{-1}\nabla |x|^{2-q}+\mathcal {O}(|x|^{2-q}\ln (|x|)),&{}q=4,\\ \frac{1}{2-q}\Vert \mathbb {S}^{(q-1)}\Vert ^{-1}\nabla |x|^{2-q}+\mathcal {O}(|x|^{2-q}),&{}q\in \mathbb {N}{\setminus }\{1,2,4\}.\end{array}\right. \end{aligned}$$
  By $\Vert \mathbb {S}^{(q-1)}\Vert$ we mean the surface area of the unit sphere $\mathbb {S}^{(q-1)}=\{x\in \mathbb {R}^q:|x|=1\}$.
(iv) The integral average vanishes, i.e., $\int _{\mathcal {F}_\Lambda }G(\Delta ;\Lambda ;y)dy=0$.

If no confusion is likely to arise, we just write $G(\Delta ;\cdot )$ instead of $G(\Delta ;\Lambda ;\cdot )$. The *m*-*th iterated* $\Lambda$-*Green function* is given by

$$\begin{aligned} G(\Delta ^m;x)=\int _{\mathcal {F}_{\Lambda }}G(\Delta ^{m-1};y)G(\Delta ;x-y)dy, \quad m\in \mathbb {N},m\ge 2, \end{aligned}$$

where $$G(\Delta ^1;\cdot )=G(\Delta ;\cdot )$$.

$G(\Delta ;\Lambda ;\cdot )$ is defined uniquely by the properties above. For $m>\frac{q}{2}$, the Green function $G(\Delta ^m;\cdot )$ is continuous in $\mathbb {R}^q$ and one can deduce

2.1 $$\begin{aligned} G(\Delta ^m;x)=\frac{1}{\Vert \mathcal {F}_\Lambda \Vert }\sum _{h\in \Lambda ^{-1}{\setminus }\{0\}}\frac{e^{2\pi i x\cdot h}}{(-4\pi ^2 h^2)^m},\quad x \in \mathbb {R}^q. \end{aligned}$$

Throughout the course of this paper, we say that $\mathcal {G}\subset \mathbb {R}^q$ is a *regular region* if it is bounded and its boundary $\partial \mathcal {G}$ is a piecewise smooth Lipschitzian manifold. The notation $\Sigma '$ that is frequently used denotes the sum

2.2 $$\begin{aligned} {\sum _{g\in \Lambda \cap \overline{\mathcal {G}}}}'F(g)= \sum _{g\in \Lambda \cap {\mathcal {G}}}F(g)+\sum _{g\in \Lambda \cap \partial {\mathcal {G}}}\alpha (g)F(g), \end{aligned}$$

where $\alpha (g)$ denotes the solid angle at a point $g\in \partial \mathcal {G}$, normalized to values in the interval [0, 1]. For example, if $\mathcal {G}$ has a $C^{(1)}$-boundary, then $\alpha (g)=\frac{1}{2}$ for all $g\in \partial \mathcal {G}$. In case $\mathcal {G}=[0,1]^3$ is a three-dimensional cube, then $\alpha (g)=\frac{1}{8}$ if *g* is a vertex, $\alpha (g)=\frac{1}{4}$ if *g* lies on an edge, and $\alpha (g)=\frac{1}{2}$ if *g* lies on a face of the cube.

Application of Green’s formulas and the properties from Definition 2.2 lead to the following Euler summation formula (for details, the reader is referred to Freeden (2011) and references therein).

### Theorem 2.3

Let $\mathcal {G}\subset \mathbb {R}^q$ be a regular region, $G(\Delta ;\cdot )$ the $\Lambda$-Green function for a lattice $\Lambda$ in $\mathbb {R}^q$, and $F\in C^{(2)}(\overline{\mathcal {G}})$. Then

$$\begin{aligned} {\sum _{g\in \Lambda \cap \overline{\mathcal {G}}}}'F(g)=&\frac{1}{\Vert \mathcal {F}_\Lambda \Vert }\int _{\mathcal {G}}F(y)dy+\int _{\mathcal {G}}G(\Delta ;y)\Delta F(y)dy\\&+\int _{\partial \mathcal {G}}\left( F(y)\frac{\partial }{\partial \nu } G(\Delta ;y)-G(\Delta ;y)\frac{\partial }{\partial \nu } F(y)\right) dS(y). \end{aligned}$$

Iterated application of Green’s formulas to Theorem 2.3 leads to the Euler summation formula for $\Delta ^m$.

### Theorem 2.4

Let $\mathcal {G}\subset \mathbb {R}^q$ be a regular region, $G(\Delta ;\cdot )$ the $\Lambda$-Green function for a lattice $\Lambda$ in $\mathbb {R}^q$, and $F\in C^{(2m)}(\overline{\mathcal {G}})$ for some $m\in \mathbb {N}$. Then

$$\begin{aligned} {\sum _{g\in \Lambda \cap \overline{\mathcal {G}}}}'F(g)=&\frac{1}{\Vert \mathcal {F}_\Lambda \Vert }\int _{\mathcal {G}}F(y)dy+\int _{\mathcal {G}}G(\Delta ^m;y)\Delta ^m F(y)dy\\&+\sum _{k=0}^{m-1}\int _{\partial \mathcal {G}}\Delta ^kF(y)\frac{\partial }{\partial \nu } G(\Delta ^{k+1};y)dS(y)\\&-\sum _{k=0}^{m-1}\int _{\partial \mathcal {G}}G(\Delta ^{k+1};y)\frac{\partial }{\partial \nu } \Delta ^kF(y)dS(y). \end{aligned}$$

For a fixed lattice $\Lambda \subset \mathbb {R}^q$, we now investigate the contracted lattice $\tau \Lambda$, $\tau \in (0,1]$, and observe that

2.3 $$\begin{aligned} G(\Delta ^m;\tau \Lambda ;x)=\tau ^{2m-q}G\left( \Delta ^m;\Lambda ;\frac{x}{\tau }\right) ,\quad x\in \mathbb {R}^q{\setminus }\tau \Lambda . \end{aligned}$$

Applying Theorem 2.4 to the contracted lattice leads to the following corollary.

### Corollary 2.5

Let $\mathcal {G}\subset \mathbb {R}^q$ be a regular region, $G(\Delta ;\cdot )$ the $\Lambda$-Green function for a lattice $\Lambda$ in $\mathbb {R}^q$, $F\in C^{(2m)}(\overline{\mathcal {G}})$ for some $m\in \mathbb {N}$, and $\tau \in (0,1]$. Then

$$\begin{aligned} \tau ^q{\sum _{ g\in \tau \Lambda \cap \overline{\mathcal {G}}}}'F( g)=&\frac{1}{\Vert \mathcal {F}_\Lambda \Vert }\int _{\mathcal {G}}F(y)dy+\tau ^{2m}\int _{\mathcal {G}}G(\Delta ^m;\frac{y}{\tau })\Delta ^m F(y)dy\\&+\sum _{k=0}^{m-1}\tau ^{2k+2}\int _{\partial \mathcal {G}}\Delta ^kF(y)\frac{\partial }{\partial \nu } G(\Delta ^{k+1};\frac{y}{\tau })dS(y)\\&-\sum _{k=0}^{m-1}\tau ^{2k+2}\int _{\partial \mathcal {G}}G(\Delta ^{k+1};\frac{y}{\tau })\frac{\partial }{\partial \nu } \Delta ^kF(y)dS(y). \end{aligned}$$

Eventually, defining the *cubature rule* $Q_{\tau }:C^{(2m)}(\overline{\mathcal {G}})\rightarrow \mathbb {R}$ with respect to $\Lambda$ and $\tau$ by

2.4 $$\begin{aligned} Q_{\tau }[F]=\tau ^q{\sum _{g\in \tau \Lambda \cap \overline{\mathcal {G}}}}'F(g)-\tau ^{2}\int _{\partial \mathcal {G}}F(y)\frac{\partial }{\partial \nu } G(\Delta ;\frac{y}{\tau })dS(y), \end{aligned}$$

the corollary above allows the following representation of the *cubature error*

2.5 $$\begin{aligned} R_{\tau }[F]=&Q_{\tau }[F]-\frac{1}{\Vert \mathcal {F}_\Lambda \Vert }\int _{\mathcal {G}}F(y)dy\nonumber \\ =&\tau ^{2m}\int _{\mathcal {G}}G(\Delta ^m;\frac{y}{\tau })\Delta ^m F(y)dy\nonumber \\&+\sum _{k=1}^{m-1}\tau ^{2k+2}\int _{\partial \mathcal {G}}\Delta ^kF(y)\frac{\partial }{\partial \nu } G(\Delta ^{k+1};\frac{y}{\tau })dS(y)\nonumber \\&-\sum _{k=0}^{m-1}\tau ^{2k+2}\int _{\partial \mathcal {G}}G(\Delta ^{k+1};\frac{y}{\tau })\frac{\partial }{\partial \nu } \Delta ^kF(y)dS(y). \end{aligned}$$

In the particular case of (2.4), the integral on the right hand side is to be understood in the sense $\int _{\partial \mathcal {G}}\ldots =\lim _{\varepsilon \rightarrow 0+}\int _{\partial \mathcal {G}{\setminus }\bigcup _{g\in \tau \Lambda }\mathbb {B}_\varepsilon (g)}\ldots$. Thus, for $q=1$ and a contracted lattice $\tau \Lambda$ such that the end points of the interval [0, *b*] coincide with points of the contracted lattice, the integral $\int _{\partial \mathcal {G}}\ldots$ vanishes and we end up with the one-dimensional trapezoidal rule (1.6). For $q\ge 2$, the interpretation $\int _{\partial \mathcal {G}}\ldots =\lim _{\varepsilon \rightarrow 0+}\int _{\partial \mathcal {G}{\setminus }\bigcup _{g\in \tau \Lambda }\mathbb {B}_\varepsilon (g)}\ldots$ leads to the standard surface integral over $\partial \mathcal {G}$.

### Remark 2.6

Since the integrals that occur on the right hand side of (2.5) are bounded uniformly in $\tau \in (0,1]$, the cubature error behaves like

2.6 $$\begin{aligned} | R_{\tau }[F]|=\mathcal {O}(\tau ^2) \end{aligned}$$

if no further conditions other than $F\in C^{(2m)}(\overline{\mathcal {G}})$, $m\ge 1$, are assumed. Under the assumption of periodicity (in the sense that the boundary integrals $\int _{\partial \mathcal {G}}\Delta ^kF(y)\frac{\partial }{\partial \nu } G(\Delta ^{k+1};\frac{y}{\tau })dS(y)=0$ and $\int _{\partial \mathcal {G}}G(\Delta ^{k+1};\frac{y}{\tau })\frac{\partial }{\partial \nu } \Delta ^kF(y)dS(y)=0$ vanish for $k=0,\ldots ,m-1$), the cubature error improves to

2.7 $$\begin{aligned} |R_{\tau }[F]|=\mathcal {O}(\tau ^{2m}). \end{aligned}$$

Latter condition, however, is hardly ever satisfied in actual applications. In the next section we, therefore, discuss how classical Romberg extrapolation (see, e.g., Antes 1972; Romberg 1955; Schönhage 1970) transfers to Euler summation based cubature on regular regions.

## Romberg extrapolation

We begin by applying the first classical Romberg step, i.e., we substitute the cubature rule $Q_\tau$ from (2.4) by the linear combination

3.1 $$\begin{aligned} \bar{Q}_{c,\tau }=\frac{Q_{c\tau }-c^2Q_\tau }{1-c^2}, \end{aligned}$$

for some $c\in (0,1)$ and $\tau \in (0,1]$. According to (2.5), the remainder term for this cubature rule reads

3.2 $$\begin{aligned} \bar{R}_{c,\tau }[F]=&\bar{Q}_{c,\tau }[F]-\frac{1}{\Vert \mathcal {F}_\Lambda \Vert }\int _{\mathcal {G}}F(y)dy\nonumber \\ =&\frac{1}{1-c^2}R_{c\tau }[F]-\frac{c^2}{1-c^2}R_{\tau }[F]\nonumber \\ =&\frac{c^2}{1-c^2}\tau ^{2m}\int _{\mathcal {G}}\left( c^{2m-2}G(\Delta ^m;\frac{y}{c\tau })-G(\Delta ^m;\frac{y}{\tau })\right) \Delta ^m F(y)dy\nonumber \\&-\sum _{k=1}^{m-1}\frac{c^2}{1-c^2}(1-c^{2k})\tau ^{2k+2}\int _{\partial \mathcal {G}}\Delta ^kF(y)\frac{\partial }{\partial \nu } G(\Delta ^{k+1};\frac{y}{\tau })dS(y)\nonumber \\&+\sum _{k=0}^{m-1}\frac{c^2}{1-c^2}(1-c^{2k})\tau ^{2k+2}\int _{\partial \mathcal {G}}G(\Delta ^{k+1};\frac{y}{\tau })\frac{\partial }{\partial \nu } \Delta ^kF(y)dS(y)\nonumber \\&+\bar{E}_{c,\tau }[F]\nonumber \\ =&\frac{c^2}{1-c^2}\tau ^{2m}\int _{\mathcal {G}}\left( c^{2m-2}G(\Delta ^m;\frac{y}{c\tau })-G(\Delta ^m;\frac{y}{\tau })\right) \Delta ^m F(y)dy\nonumber \\&-\sum _{k=1}^{m-1}\frac{c^2}{1-c^2}(1-c^{2k})\tau ^{2k+2}\int _{\partial \mathcal {G}}\Delta ^kF(y)\frac{\partial }{\partial \nu } G(\Delta ^{k+1};\frac{y}{\tau })dS(y)\nonumber \\&+\sum _{k=1}^{m-1}\frac{c^2}{1-c^2}(1-c^{2k})\tau ^{2k+2}\int _{\partial \mathcal {G}}G(\Delta ^{k+1};\frac{y}{\tau })\frac{\partial }{\partial \nu } \Delta ^kF(y)dS(y)\nonumber \\&+\bar{E}_{c,\tau }[F], \end{aligned}$$

where

3.3 $$\begin{aligned} \bar{E}_{c,\tau }[F]=&\sum _{k=1}^{m-1}\frac{(c\tau )^{2k+2}}{1-c^2}\int _{\partial \mathcal {G}}\Delta ^kF(y)\frac{\partial }{\partial \nu } D(\Delta ^{k+1};\frac{y}{\tau })dS(y)\nonumber \\&-\sum _{k=0}^{m-1}\frac{(c\tau )^{2k+2}}{1-c^2}\int _{\partial \mathcal {G}}D(\Delta ^{k+1};\frac{y}{\tau })\frac{\partial }{\partial \nu } \Delta ^kF(y)dS(y), \end{aligned}$$

and $D(\Delta ^{k};x)=G(\Delta ^{k};\frac{x}{c})-G(\Delta ^{k};x)$. Equation (3.2) shows that, for functions $F\in C^{(2m)}(\overline{\mathcal {G}})$, $m\ge 2$, the convergence rate of the modified cubature rule *Q* from (3.1) is

3.4 $$\begin{aligned} |\bar{R}_{c,\tau }[F]|=|\bar{E}_{c,\tau }[F]|+\mathcal {O}(\tau ^{4}) \end{aligned}$$

while the convergence rates for the original cubature rules $Q_\tau$ and $Q_{c\tau }$ are $|R_\tau [F]|=\mathcal {O}(\tau ^{2})$ and $|R_{c\tau }[F]|=\mathcal {O}(\tau ^{2})$, respectively, according to Remark 2.6. Therefore, it remains to investigate the term $\bar{E}_{c,\tau }[F]$.

### Remark 3.1

In order to indicate the relation to the classical Romberg scheme, we return to the setup of the introductory Sect. 1.1 with $q=1$, $m=2$, and $\mathcal {G}=[0,b]$ (i.e., the boundary $\partial \mathcal {G}$ contains only the points 0, *b*). We observe that the $\Lambda$-Green function $G(\Delta ;\Lambda ;\cdot )$ and the iterated $\Lambda$-Green function $G(\Delta ^2;\Lambda ;\cdot )$ for the lattice $\Lambda =\mathbb {Z}$ are given as in (1.3) and (1.4), respectively. Furthermore, we choose $\tau =\frac{b}{n}$ and $c=\frac{1}{p}$, with fixed numbers $n,p\in \mathbb {N}$. Then $\frac{b}{\tau },\frac{b}{c\tau }\in \mathbb {Z}$, which implies that $b\in \tau \mathbb {Z}$, $b\in c\tau \mathbb {Z}$. Since boundary integrals in the one-dimensional setting are just point evaluations in the end points 0 and *b*, we obtain from Eq. (3.3) that

3.5 $$\begin{aligned} \bar{E}_{c,\tau }[F]=&-\frac{(c\tau )^{2}}{1-c^2}\underbrace{\left[ D(\Delta ;\frac{y}{\tau })\frac{\partial }{\partial y} F(y)\right] _{y=0}^{y=b}}_{=0} +\frac{(c\tau )^{4}}{1-c^2}\underbrace{\left[ \frac{\partial ^2}{\partial y^2} F(y)\frac{\partial }{\partial y} D(\Delta ^2;\frac{y}{\tau })\right] _{y=0}^{y=b}}_{=0}\nonumber \\&-\frac{(c\tau )^{4}}{1-c^2}\underbrace{\left[ D(\Delta ^2;\frac{y}{\tau })\frac{\partial ^3}{\partial y^3} F(y)\right] _{y=0}^{y=b}}_{=0} =0. \end{aligned}$$

In other words, the cubature rule $\bar{Q}_{c,\tau }[F]$ satisfies the improved convergence rate $|\bar{R}_{c,\tau }[F]|=\mathcal {O}(\tau ^4)$, as already seen in Sect. 1.1.

Remark 3.1 tells us that the boundary term $\bar{E}_{c,\tau }$ vanishes in the one-dimensional setting. Unfortunately, this is not true for $\bar{E}_{c,\tau }$ in the general *q*-dimensional case and the boundary terms have an influence on the modified cubature rule $Q_{c,\tau }=\bar{Q}_{c,\tau }+\bar{E}_{c,\tau }$, which eventually satisfies $R_{c,\tau }[F]=Q_{c,\tau }[F]-\frac{1}{\Vert \mathcal {F}_\Lambda \Vert }\int _{\mathcal {G}}F(y)dy=\mathcal {O}(\tau ^4)$.

A further improvement of the convergence rate to $\mathcal {O}(\tau ^{2\ell })$ requires an iteration of the Romberg step. We make the following more general definitions.

### Definition 3.2

Let $\bar{Q}_i^{(0)}$, $i\in \mathbb {N}_0$, denote the cubature rule $Q_{c^i\tau }$ from (2.4) with respect to the lattice $\Lambda$ and $c^i\tau$, where $c\in (0,1)$ and $\tau \in (0,1]$ are fixed. Then, we iteratively define new cubature rules by

$$\begin{aligned} \bar{Q}_i^{(\ell )}=&\frac{\bar{Q}_i^{(\ell -1)}-c^{2\ell }\bar{Q}_{i-1}^{(\ell -1)}}{1-c^{2\ell }}, \quad \ell \in \mathbb {N}, i=\ell ,\ell +1\,\ldots . \end{aligned}$$

Additionally, let $\bar{E}_i^{(0)}=0$, $i\in \mathbb {N}_0$. Then, we iteratively define correction terms for $\ell \in \mathbb {N}$, $i=\ell ,\ell +1,\ldots$, by

$$\begin{aligned} \bar{E}_i^{(\ell )}[F]=&\frac{\bar{E}_i^{(\ell -1)}[F]-c^{2\ell }\bar{E}_{i-1}^{(\ell -1)}[F]}{1-c^{2\ell }}\\&+(-1)^{\ell -1}\Bigg (\sum _{k=\max \{1,\ell -1\}}^{m-1}\frac{(c^{i-(\ell -1)}\tau )^{2k+2}\prod _{j=1}^{\ell -1}c^{2j}}{\prod _{j=1}^{\ell }(1-c^{2j})}\left( \prod _{j=0}^{\ell -2}(1-c^{2(k-j)})\right) \\&\times \int _{\partial \mathcal {G}}\Delta ^kF(y)\frac{\partial }{\partial \nu } D\left( \Delta ^{k+1};\frac{y}{c^{i-\ell }\tau }\right) dS(y)\Bigg )\\&-(-1)^{\ell -1}\Bigg (\sum _{k=\ell -1}^{m-1}\frac{(c^{i-(\ell -1)}\tau )^{2k+2}\prod _{j=1}^{\ell -1}c^{2j}}{\prod _{j=1}^{\ell }(1-c^{2j})}\left( \prod _{j=0}^{\ell -2}(1-c^{2(k-j)})\right) \\&\times \int _{\partial \mathcal {G}}D\left( \Delta ^{k+1};\frac{y}{c^{i-\ell }\tau }\right) \frac{\partial }{\partial \nu } \Delta ^kF(y)dS(y)\Bigg ), \end{aligned}$$

where, again, $$D(\Delta ^{k};x)=G(\Delta ^{k};\frac{x}{c})-G(\Delta ^{k};x)$$.

In particular, $\bar{Q}_1^{(1)}$ denotes the first Romberg step $\bar{Q}_{c,\tau }$ as indicated in (3.1) and $\bar{E}_1^{(1)}$ denotes the correction term $\bar{E}_{c,\tau }$ as indicated in (3.3). For the iterates $\bar{Q}_i^{(\ell )}$, we obtain the following expression of the remainder term $\bar{R}_i^{(\ell )}$.

### Theorem 3.3

Let $\mathcal {G}\subset \mathbb {R}^q$ be a regular region, $G(\Delta ;\cdot )$ the $\Lambda$-Green function for a lattice $\Lambda$ in $\mathbb {R}^q$, and $F\in C^{(2m)}(\overline{\mathcal {G}})$ for some $m\in \mathbb {N}$. Then, for $\ell =1,\ldots ,m-1$, $i=\ell ,\ell +1,\ldots$,

3.6 $$\begin{aligned} \bar{R}_i^{(\ell )}[F]=&\bar{Q}_i^{(\ell )}[F]-\frac{1}{\Vert \mathcal {F}_\Lambda \Vert }\int _{\mathcal {G}}F(y)dy\nonumber \\ =&\frac{(c^{i-\ell })^{2m}\prod _{j=1}^{\ell }c^{2j}}{\prod _{j=1}^{\ell }(1-c^{2j})}\tau ^{2m}\int _{\mathcal {G}}\left( \sum _{j=0}^\ell a_j^{(\ell )}G\left( \Delta ^m;\frac{y}{c^{i-\ell +j}\tau }\right) \right) \Delta ^mF(y)dy\nonumber \\&+(-1)^\ell \Bigg [\sum _{k=\ell }^{m-1}\frac{(c^{i-\ell })^{2k+2}\prod _{j=1}^{\ell }c^{2j}}{\prod _{j=1}^{\ell }(1-c^{2j})}\left( \prod _{j=0}^{\ell -1}(1-c^{2(k-j)})\right) \nonumber \\&\times \tau ^{2k+2}\int _{\partial \mathcal {G}}\Delta ^kF(y)\frac{\partial }{\partial \nu } G\left( \Delta ^{k+1};\frac{y}{c^{i-\ell }\tau }\right) dS(y)\Bigg ] \nonumber \\&-(-1)^\ell \Bigg [\sum _{k=\ell }^{m-1}\frac{(c^{i-\ell })^{2k+2}\prod _{j=1}^{\ell }c^{2j}}{\prod _{j=1}^{\ell }(1-c^{2j})}\left( \prod _{j=0}^{\ell -1}(1-c^{2(k-j)})\right) \nonumber \\&\times \tau ^{2k+2}\int _{\partial \mathcal {G}} G\left( \Delta ^{k+1};\frac{y}{c^{i-\ell }\tau }\right) \frac{\partial }{\partial \nu }\Delta ^kF(y)dS(y)\Bigg ] +\bar{E}_i^{(\ell )}[F]. \end{aligned}$$

The coefficients $a_j^{(\ell )}$ are iteratively defined via $a_0^{(\ell )}=(-1)^\ell$, $a_j^{(\ell )}=c^{2(m-\ell )}a_{j-1}^{(\ell -1)}-a_j^{(\ell -1)}$, $j=1,\ldots ,\ell$, and $a_j^{(\ell )}=0$, $j=\ell +1,\ell +2,\ldots$.

### Proof

For $\ell =1$, the assertion can be checked by hand. Now, assuming that the representation (3.6) of $\bar{R}_i^{(\ell )}[F]$ is true for $\ell$ and all $i=\ell ,\ell +1,\ldots$, we show by induction that it also holds true for $\ell +1$ and all $i=\ell +1,\ell +2,\ldots$. First, we observe

3.7 $$\begin{aligned} \bar{R}_i^{(\ell +1)}[F]=&\frac{1}{1-c^{2\ell +2}}\bar{R}_i^{(\ell )}[F]-\frac{c^{2\ell +2}}{1-c^{2\ell +2}}\bar{R}_{i-1}^{(\ell )}[F]. \end{aligned}$$

For the explicit computation of the right hand side of Eq. (3.7), we treat the different terms of $\bar{R}_i^{(\ell )}[F]$ and $\bar{R}_{i-1}^{(\ell )}[F]$, as appearing on the right hand side of Eq. (3.6), separately. For the first summand, we obtain

$$\begin{aligned}&\frac{1}{1-c^{2\ell +2}}\frac{(c^{i-\ell })^{2m}\prod _{j=1}^{\ell }c^{2j}}{\prod _{j=1}^{\ell }(1-c^{2j})}\tau ^{2m}\int _{\mathcal {G}}\left( \sum _{j=0}^\ell a_j^{(\ell )}G\left( \Delta ^m;\frac{y}{c^{i-\ell +j}\tau }\right) \right) \Delta ^mF(y)dy\\&\qquad -\frac{c^{2\ell +2}}{1-c^{2\ell +2}}\frac{(c^{i-1-\ell })^{2m}\prod _{j=1}^{\ell }c^{2j}}{\prod _{j=1}^{\ell }(1-c^{2j})}\tau ^{2m}\int _{\mathcal {G}}\left( \sum _{j=0}^\ell a_j^{(\ell )}G\left( \Delta ^m;\frac{y}{c^{i-1-\ell +j}\tau }\right) \right) \Delta ^mF(y)dy\\&\quad =\frac{(c^{i-(\ell +1)})^{2m}\prod _{j=1}^{\ell }c^{2j}}{\prod _{j=1}^{\ell +1}(1-c^{2j})}\tau ^{2m}\int _{\mathcal {G}}\Bigg [c^{2m}\sum _{j=0}^\ell a_j^{(\ell )}G\left( \Delta ^m;\frac{y}{c^{i-\ell +j}\tau }\right) \\&\qquad -c^{2\ell +2}\sum _{j=0}^\ell a_j^{(\ell )}G\left( \Delta ^m;\frac{y}{c^{i-(\ell +1)+j}\tau }\right) \Bigg ]\Delta ^mF(y)dy\\&\quad =\frac{(c^{i-(\ell +1)})^{2m}\prod _{j=1}^{\ell +1}c^{2j}}{\prod _{j=1}^{\ell +1}(1-c^{2j})}\tau ^{2m}\int _{\mathcal {G}}\Bigg [c^{2m-2(\ell +1)}\sum _{j=0}^\ell a_j^{(\ell )}G\left( \Delta ^m;\frac{y}{c^{i-\ell +j}\tau }\right) \\&\qquad -\sum _{j=0}^\ell a_j^{(\ell )}G\left( \Delta ^m;\frac{y}{c^{i-(\ell +1)+j}\tau }\right) \Bigg ]\Delta ^mF(y)dy\\&\quad =\frac{(c^{i-(\ell +1)})^{2m}\prod _{j=1}^{\ell +1}c^{2j}}{\prod _{j=1}^{\ell +1}(1-c^{2j})}\tau ^{2m}\int _{\mathcal {G}}\Bigg [-a_0^{(\ell )}G\left( \Delta ^m;\frac{y}{c^{i-(\ell +1)}\tau }\right) \\&\qquad +\sum _{j=1}^{\ell +1} \left( c^{2(m-(\ell +1))}a_{j-1}^{(\ell )}-a_{j}^{(\ell )}\right) G\left( \Delta ^m;\frac{y}{c^{i-(\ell +1)+j}\tau }\right) \Bigg ]\Delta ^mF(y)dy. \end{aligned}$$

The representation of the coefficients $a_j^{(\ell +1)}$ can be obtained directly from the right hand side of the equation above. For the second summand of the right hand side of Eq. (3.6), we get

$$\begin{aligned}&\frac{1}{1-c^{2(\ell +1)}}\sum _{k=\ell }^{m-1}\Bigg [\frac{(c^{i-\ell })^{2k+2}\prod _{j=1}^{\ell }c^{2j}}{\prod _{j=1}^{\ell }(1-c^{2j})}\left( \prod _{j=0}^{\ell -1}(1-c^{2(k-j)})\right) \\&\qquad \times \tau ^{2k+2}\int _{\partial \mathcal {G}}\Delta ^kF(y)\frac{\partial }{\partial \nu } G\left( \Delta ^{k+1};\frac{y}{c^{i-\ell }\tau }\right) dS(y)\Bigg ]\\&\qquad -\frac{c^{2(\ell +1)}}{1-c^{2(\ell +1)}}\sum _{k=\ell }^{m-1}\Bigg [\frac{(c^{i-1-\ell })^{2k+2}\prod _{j=1}^{\ell }c^{2j}}{\prod _{j=1}^{\ell }(1-c^{2j})}\left( \prod _{j=0}^{\ell -1}(1-c^{2(k-j)})\right) \\&\qquad \times \tau ^{2k+2}\int _{\partial \mathcal {G}}\Delta ^kF(y)\frac{\partial }{\partial \nu } G\left( \Delta ^{k+1};\frac{y}{c^{i-1-\ell }\tau }\right) dS(y)\Bigg ]\\&\quad =\sum _{k=\ell }^{m-1}\Bigg [\left( \frac{c^{2k+2}}{1-c^{2(\ell +1)}}-\frac{c^{2(\ell +1)}}{1-c^{2(\ell +1)}}\right) \frac{(c^{i-1-\ell })^{2k+2}\prod _{j=1}^{\ell }c^{2j}}{\prod _{j=1}^{\ell }(1-c^{2j})}\left( \prod _{j=0}^{\ell -1}(1-c^{2(k-j)})\right) \\&\qquad \times \tau ^{2k+2}\int _{\partial \mathcal {G}}\Delta ^kF(y)\frac{\partial }{\partial \nu } G\left( \Delta ^{k+1};\frac{y}{c^{i-1-\ell }\tau }\right) dS(y)\Bigg ]\\&\qquad +\frac{1}{1-c^{2(\ell +1)}}\sum _{k=\ell }^{m-1}\Bigg [\frac{(c^{i-\ell })^{2k+2}\prod _{j=1}^{\ell }c^{2j}}{\prod _{j=1}^{\ell }(1-c^{2j})}\left( \prod _{j=0}^{\ell -1}(1-c^{2(k-j)})\right) \\&\qquad \times \tau ^{2k+2}\int _{\partial \mathcal {G}}\Delta ^kF(y)\frac{\partial }{\partial \nu } D\left( \Delta ^{k+1};\frac{y}{c^{i-1-\ell }\tau }\right) dS(y)\Bigg ]\\&\quad =-\sum _{k=\ell +1}^{m-1}\Bigg [\frac{(c^{i-(\ell +1)})^{2k+2}\prod _{j=1}^{\ell +1}c^{2j}}{\prod _{j=1}^{\ell +1}(1-c^{2j})}\left( \prod _{j=0}^{\ell }(1-c^{2(k-j)})\right) \\&\qquad \underbrace{\times \tau ^{2k+2}\int _{\partial \mathcal {G}}\Delta ^kF(y)\frac{\partial }{\partial \nu } G\left( \Delta ^{k+1};\frac{y}{c^{i-(\ell +1)}\tau }\right) dS(y)\Bigg ]}_{\text {part of }\bar{R}_i^{(\ell +1)}[F]\text { according to equation (3.6)}}\\&\qquad +\sum _{k=\ell }^{m-1}\Bigg [\frac{(c^{i-\ell })^{2k+2}\prod _{j=1}^{\ell }c^{2j}}{\prod _{j=1}^{\ell +1}(1-c^{2j})}\left( \prod _{j=0}^{\ell -1}(1-c^{2(k-j)})\right) \\&\qquad \underbrace{\times \tau ^{2k+2}\int _{\partial \mathcal {G}}\Delta ^kF(y)\frac{\partial }{\partial \nu } D\left( \Delta ^{k+1};\frac{y}{c^{i-(\ell +1)}\tau }\right) dS(y)\Bigg ]}_{\text {part of }\bar{E}_i^{(\ell +1)}[F]\text { according to equation (3.6) and Definition 3.2}}. \end{aligned}$$

The contributions of the third term of the right hand side of Eq. (3.6) can be reformulated very analogously and the fourth term simply yields

3.8 $$\begin{aligned} \frac{1}{1-c^{2\ell +2}}\bar{E}_i^{(\ell )}[F]-\frac{c^{2\ell +2}}{1-c^{2\ell +2}}\bar{E}_{i-1}^{(\ell )}[F]. \end{aligned}$$

Combining all computations above via (3.7) and observing Definition 3.2 leads to the desired representation of $\bar{R}_i^{(\ell +1)}[F]$. $\square$

### Corollary 3.4

Let $\mathcal {G}\subset \mathbb {R}^q$ be a regular region, $G(\Delta ;\cdot )$ the $\Lambda$-Green function for a lattice $\Lambda$ in $\mathbb {R}^q$, and $F\in C^{(2m)}(\overline{\mathcal {G}})$ for some $m\in \mathbb {N}$. Then, for $\ell =1,\ldots ,m-1$, $i=\ell ,\ell +1,\ldots$, the cubature rule $Q_i^{(\ell )}=\bar{Q}_i^{(\ell )}-\bar{E}_i^{(\ell )}$ satisfies

$$\begin{aligned} \left| R_i^{(\ell )}[F]\right| =&\left| Q_i^{(\ell )}[F]-\frac{1}{\Vert \mathcal {F}_\Lambda \Vert }\int _{\mathcal {G}}F(y)dy\right| =\mathcal {O}(\tau ^{2\ell +2}). \end{aligned}$$

**Fig. 1:**
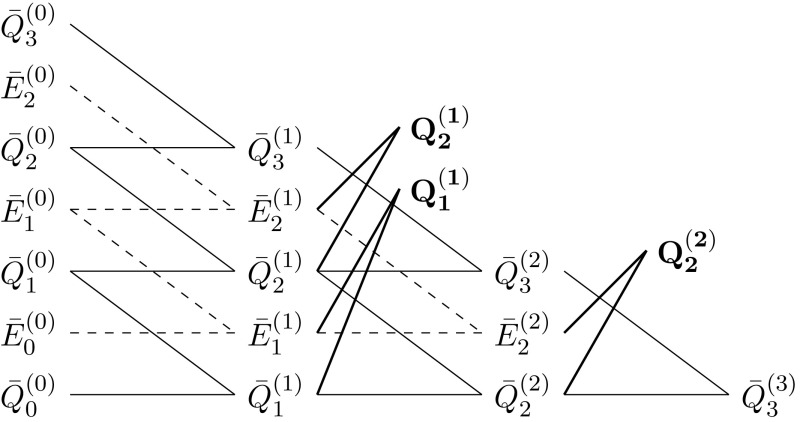
Illustration of the iterative construction of $Q_i^{(\ell )}$

Corollary 3.4 states the desired improved convergence rate for the Romberg extrapolated cubature rule $Q_i^{(\ell )}$ that holds true for general regular regions $\mathcal {G}\subset \mathbb {R}^q$. An illustration of the iterative construction of the cubature rule can be found in Fig. 1.

### Remark 3.5

In the case of one-dimensional numerical integration, we proceed as in Remark 3.1 and choose the interval [0, *b*], $\tau =\frac{b}{n}$, and $c=\frac{1}{p}$, with fixed numbers $n,p\in \mathbb {N}$. However, this time we do not investigate the first Romberg step but we look at its $\ell$th iterate for $\ell \ge 2$. Then, the term $\bar{E}_{\ell }^{(\ell )}$ from Definition 3.2 becomes

3.9 $$\begin{aligned} \bar{E}_{\ell }^{(\ell )}[F]=&\frac{\bar{E}_{\ell -1}^{(\ell )}[F]-c^{2\ell }\bar{E}_{\ell -1}^{(\ell -1)}[F]}{1-c^{2\ell }}\nonumber \\&+(-1)^{\ell -1}\sum _{k=\ell -1}^{m-1}\Bigg (\frac{\tau ^{2k+2}\prod _{j=1}^{\ell -1}c^{2j}}{\prod _{j=1}^{\ell }(1-c^{2j})}\left( \prod _{j=0}^{\ell -2}(1-c^{2(k-j)})\right) \nonumber \\&\times \underbrace{\left[ \Delta ^kF(y)\frac{\partial }{\partial \nu } D\left( \Delta ^{k+1};\frac{y}{\tau }\right) \right] _{y=0}^{y=b}}_{=0}\Bigg )\nonumber \\&-(-1)^{\ell -1}\sum _{k=\ell -1}^{m-1}\Bigg (\frac{\tau ^{2k+2}\prod _{j=1}^{\ell -1}c^{2j}}{\prod _{j=1}^{\ell }(1-c^{2j})}\left( \prod _{j=0}^{\ell -2}(1-c^{2(k-j)})\right) \nonumber \\&\times \underbrace{\left[ D\left( \Delta ^{k+1};\frac{y}{\tau }\right) \frac{\partial }{\partial \nu } \Delta ^kF(y)\right] _{y=0}^{y=b}}_{=0}\Bigg )\nonumber \\ =&\frac{\bar{E}_{\ell }^{(\ell -1)}[F]-c^{2\ell }\bar{E}_{\ell -1}^{(\ell -1)}[F]}{1-c^{2\ell }}, \end{aligned}$$

for $m\ge 3$ and $\ell \ge 2$. Observing that $\bar{E}_{1}^{(1)}[F]=\bar{E}_{c,\tau }[F]=0$ in Remark 3.1, we iteratively see that $\bar{E}_{\ell }^{(\ell )}[F]$ vanishes and obtain the classical one-dimensional Romberg method $Q_\ell ^{(\ell )}=\bar{Q}_\ell ^{(\ell )}$ with convergence rate $\mathcal {O}(\tau ^{2\ell })$.

## Conclusion

We have provided an explicit expression for a Romberg scheme on general regular regions in $\mathbb {R}^q$ that achieves the convergence rate known for the one-dimensional case and specific geometries like cuboids in higher dimensions. The cubature formulas $Q_i^{(\ell )}$ involve the $\Lambda$-Green function $G(\Delta ;\Lambda ;\cdot )$ for which there are no closed representations and no uniformly and absolutely convergent series representations available in dimensions $q\ge 2$. However, ball-averaged or Gauss-averaged Green’s functions as described in Freeden (2011), Freeden and Ostermann (2013) might remedy this issue. A more detailed study of the interplay of the averaging parameter and the grid size $\tau$ could help to obtain efficient evaluation methods for $\Lambda$-Green functions that allow to make use of the improved convergence rates for $Q_i^{(\ell )}$. The goal of this paper was to derive the framework for an improved cubature rule on general regular regions $\mathcal {G}\subset \mathbb {R}^q$.

## References

[CR1] Antes H (1972). Über die Romberg-integration in $$n$$ dimensionen. Computing.

[CR2] Caflisch RE (1998). Monte Carlo and quasi-Monte Carlo methods. Acta Numer..

[CR3] Cools R (1997). Constructing cubature formulae: the science behind the art. Acta Numer..

[CR4] Dick J, Kuo F, Sloan IH (2013). High-dimensional integration: the quasi-Monte Carlo way. Acta Numer..

[CR5] Freeden W (1982). Multidimensional Euler summation formulas and numerical cubature. Int. Ser. Numer. Math..

[CR6] Freeden W (2011). Metaharmonic Lattice Point Theory.

[CR7] Freeden W, Fleck J (1987). Numerical integration by means of adapted Euler summation formulas. Numer. Math..

[CR8] Freeden W, Ostermann I (2013). Integration on three-dimensional regular regions based on (modified) Euler sumation. Numer. Funct. Anal. Appl..

[CR9] Lyness JN (1989). An introduction to lattice rules and their generator matrices. IMA J. Numer. Anal..

[CR10] Romberg W (1955). Vereinfachte numerische integration. Det Kongelige Norske Videnskabers Selskab Forhandlinger.

[CR11] Schönhage A (1970). Mehrdimensionale Romberg-integration. Numer. Math..

[CR12] Sloan IH, Joe S (1994). Lattice Methods for Multiple Integration.

